# A Case Study and Concise Literature Review: Adult Patient’s Initial Manifestation of Complicated Acute Otitis Media Presenting as Jugular Foramen Syndrome

**DOI:** 10.3390/clinpract15020034

**Published:** 2025-02-12

**Authors:** Sabri El-Saied, Oren Ziv, Aviad Sapir, Daniel Yafit, Daniel M. Kaplan

**Affiliations:** Department of Otolaryngology—Head & Neck Surgery, Soroka University Medical Center, Ben-Gurion University of the Negev, Beer-Sheva 8410101, Israel; orenziv@clalut.org.il (O.Z.); aviadsa1@clalit.org.il (A.S.); danielyafit@clalit.il (D.Y.); dankap@bgu.ac.il (D.M.K.)

**Keywords:** acute otitis media, cranial nerve, hoarseness, neural injury, jugular foramen syndrome

## Abstract

**Background:** Jugular foramen syndrome (JFS) is a rare condition characterized by the compression or impairment of one or more terminal cranial nerves passing through the jugular foramen. Although malignancies are the primary cause of JFS. **Methods:** In this report, we present the first documented case of JFS caused by acute otitis media in an adult patient. **Results:** A 74-year-old woman presented with ear pain, hoarseness, dysphagia, dizziness, tinnitus, and hearing loss. A physical examination revealed a reddish-bulging tympanic membrane, left-sided hearing loss, right uvula deviation, and cranial nerve palsies affecting the ninth and tenth nerves. Imaging studies confirmed temporal bone inflammation, thrombosis of the sigmoid sinus extending into the internal jugular vein, and signs of thrombophlebitis of the jugular vein. The patient underwent a cortical mastoidectomy, sigmoid sinus decompression, and ventilation tube insertion, along with antibiotic, steroid, and anticoagulant therapy. Postoperatively, the patient’s condition improved significantly. **Conclusions:** This case highlights the importance of considering complicated acute otitis media in the differential diagnosis of neurological abnormalities associated with JFS. A thorough evaluation of the patient’s medical history and radiological imaging can assist in identifying the cause of the symptoms and guide appropriate surgical or conservative treatment. Further research is essential to gain more comprehensive insights into the pathophysiology and therapeutic interventions of JFS affecting the ears.

## 1. Introduction

Jugular foramen syndrome (JFS) is a complex clinical condition that manifests when cranial nerves IX (glossopharyngeal), X (vagus), and XI (accessory) are affected as they traverse the jugular foramen, a critical aperture at the base of the skull. Owing to the confined space within this foramen, these nerves are particularly vulnerable to various pathological processes, resulting in diverse clinical manifestations [1]. The severity of symptoms is directly correlated with the extent of nerve involvement and underlying etiology. Mild presentations may include subtle dysphagia (difficulty in swallowing), alterations in vocal quality (hoarseness or breathiness), and subtle weakness affecting the palate or vocal cords. However, in severe cases, it can result in potentially life-threatening complications. These complications may include severe dysphagia, leading to aspiration pneumonia, respiratory distress due to vocal cord paralysis and, in certain cases, mortality. Therefore, an accurate and timely diagnosis is crucial to ensure effective treatment [2].

The etiological landscape of JFS is diverse and multifaceted and encompasses both benign and malignant processes. Neoplastic lesions constitute a significant category of causative agents, and paragangliomas are highly vascularized neoplasms originating from paraganglian cells within the jugular foramen. Metastatic diseases, particularly head and neck malignancies, can infiltrate the jugular foramen and its contents. In addition to neoplastic etiologies, inflammatory conditions such as infections (bacterial, viral, or fungal) or granulomatous diseases can lead to localized inflammation and edema, resulting in the compression of the adjacent cranial nerves. Infectious processes may originate from the direct extension of neighboring infections (e.g., otitis media) or through hematogenous dissemination. Granulomatous diseases such as tuberculosis or sarcoidosis can induce similar inflammatory changes. Finally, trauma, particularly involving skull base fractures affecting the jugular foramen, is another significant cause of direct nerve damage or compression [3].

Despite the well-established association between JFS and its primary causes, the relationship between JFS and otological disorders remains unclear. Although a limited number of isolated case reports have described the involvement of adjacent structures (such as the temporal bone or middle ear) in JFS pathogenesis, the direct causal relationship between common ear pathologies (such as acute or chronic otitis media, cholesteatoma, or mastoiditis) and JFS remains unclear and insufficiently documented. In a single case report, cholesteatoma extended into the intracranial region following the erosion of the temporal bone, subsequently leading to the development of JFS [4]. Furthermore, several instances of skull base osteomyelitis resulting in JFS have been documented, including cases associated with middle ear adenoma [5,6]. Acute otitis media (AOM) is a common middle-ear infection in young children. It is characterized by the rapid-onset inflammation of the middle ear and is often accompanied by upper respiratory tract infections. This condition can lead to various complications ranging from mild to severe and can be categorized into extracranial and intracranial groups. Extracranial complications include mastoiditis, the most common complication involving the spread of infection to the mastoid air cells; facial nerve paralysis due to inflammation or direct pressure on the facial nerve; and labyrinthitis, which is inflammation of the inner ear. Other extracranial complications include subperiosteal abscesses, petrositis affecting the petrous part of the temporal bone, and a rare Bezold abscess, where infection erodes the mastoid. Although less common, intracranial complications can be severe and potentially life threatening. These include meningitis, brain abscesses, lateral sinus thrombosis, and epidural abscesses. The development of such complications underscores the importance of the prompt and appropriate treatment of AOM. The early recognition and management of AOM can significantly reduce the risk of these complications. A potential association exists between acute otitis media (AOM) and jugular foramen syndrome. Nevertheless, this is the first report of otitis media complications resulting in the onset of JFS, which significantly expands our understanding of JFS etiology and underscores the critical need for enhanced clinical vigilance and comprehensive diagnostic evaluation in patients presenting with cranial nerve palsies, particularly in those with a history of otological disorders. The present case emphasizes the need for further research to elucidate the spectrum of potential associations between JFS and auricular conditions.

## 2. Methods

This study focused on patients with JFS and complicated acute otitis media at a tertiary medical center.

We implemented a comprehensive approach to data collection using computerized medical records to obtain the laboratory, imaging, and pathological data. This methodology facilitated a thorough examination of the cases and provided a detailed analysis of the patients’ conditions and treatment outcomes. The study’s exemption from institutional ethics committee approval suggests that it was considered low risk or involved retrospective data analysis.

A comprehensive literature review is also conducted. Multiple databases, including PubMed, SCOPUS, and Google Scholar were used to ensure a thorough and extensive search. The keywords used in the search were carefully selected to identify the relevant studies that focused on JFS and its associations with external, middle, and mastoid pathologies. This comprehensive literature review identified three cases in the existing literature that described various ear pathologies (external and middle ear) leading to JFS. These cases are presented and analyzed in this study.

## 3. Case Presentation

**Figure 1:**
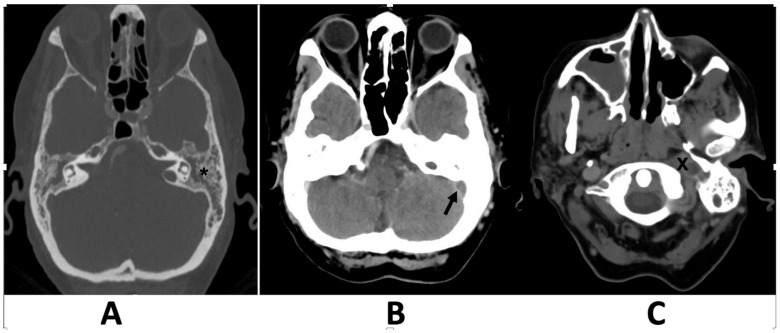
Computed tomography scans axial plane, obtained with and without contrast media administration at the level of the mastoid (**A**), showing complete opacification of the middle ear and mastoid without mastoid septal erosion (asterisk), and at the level of the sigmoid sinus showing a thrombotic lateral sinus (**B**), and the left jugular vein (**C**). A filling defect in the left sigmoid sinus (arrow) and jugular bulb (X), indicating acute thrombosis, is evident.

A 74-year-old woman visited the emergency department because of symptoms that had persisted for several weeks. These symptoms include ear pain, hoarseness, difficulty swallowing (dysphagia), dizziness, ear ringing (tinnitus), and hearing loss. Physical examination revealed a reddish bulging of the tympanic membrane. Left-sided hearing loss and right uvula deviation from the left palate paresis were also observed. The findings of the head impulse test were indicative of a positive result on the left side, whereas the examination of spontaneous horizontal nystagmus revealed its presence on the right side. Neurological examination revealed cranial nerve palsies affecting the ninth and tenth cranial nerves, respectively. Nasopharyngeal endoscopy revealed adenoid tissue that was not suspected. The blood cell counts revealed moderate neutrophilic granulocytosis and an elevated C-reactive protein level. Bacterial cultures from the ear discharge were positive for *Fusobacterium necrophorum*. High-resolution computed tomography (CT) of the left ear revealed coalescent mastoiditis and complete middle ear opacification without erosion of the mastoid septa (Figure 1).

**Figure 2:**
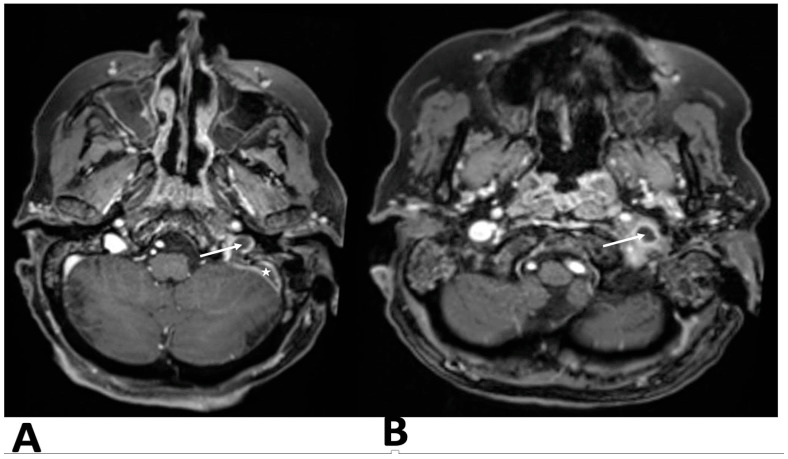
T2-weighted axial section at the level of the jugular foramen hyperintense inflammatory tissue in the left mastoid. (**A**)The flow void is absent in sigmoid sinus (asterisk) and the jugular bulb (arrow), which is hypoplastic compared with the contralateral side, JV thrombosis with EJV thrombophlebitis (**B**).

After contrast administration, the left jugular bulb and sigmoid sinus were not opacified, suggesting acute thrombosis at the proximal end of the jugular vein and sigmoid sinus. Brain and skull base magnetic resonance imaging (MRI) and magnetic resonance venography (MRV) confirmed temporal bone inflammation, sigmoid sinus thrombosis extending to the internal jugular vein, jugular vein thrombophlebitis, and no other intracranial complications (Figure 2).

The patient underwent a surgical procedure involving cortical mastoidectomy, sigmoid sinus decompression, and tympanic membrane ventilation tube insertion. During surgery, granulation tissue was discovered in the middle ear and mastoid cavity along with pus in the perisinus area. Postsurgical treatment included the administration of broad-spectrum parenteral antibiotics, steroids, and anticoagulants. Nerve paralysis in patients IX and X improved three days post-surgery, and the patient was discharged ten days after hospitalization, showing significant improvement.

## 4. Discussion

JFS is a rare condition that occurs due to compression or infiltration of structures at the base of the skull. The symptoms experienced depend on the affected structures, such as the cranial nerves and internal jugular vein. Ear involvement can lead to hearing loss, tinnitus, dizziness, and otalgia. Erol et al. (2005) described a case of middle ear cholesteatoma causing JFS, with sudden hoarseness and hearing loss. It is important to note that the symptoms of JFS can vary depending on the nerve involvement and the underlying cause [4]. Breton et al. (2021) reported a case of JFS in a patient with middle ear adenoma involving the opening of the jugular foramen. The patient presented with hearing loss and ear hemorrhage, which were initially attributed to a middle ear pathology. However, further examination revealed the presence of a jugular paraganglioma [5]. Bond et al. (2022) presented a clinical case of malignant otitis externa with Collet–Sicard syndrome. The patient had unilateral palsy of the inferior cranial nerves IX, X, XI, and XII, resulting from lesions at the skull base affecting the jugular foramen [5]. The association between JFS and venous thrombosis may be explained by the presence of infection that causes vascular damage. When a septic focus occupies a vein, the adventitia is initially affected, then it becomes obstructed and infiltrates the inflammatory cells. This inflammation then spreads throughout all the vein layers, ultimately reaching the intima, where thrombus formation occurs owing to the adhesion of fibrin, blood cells, and platelets [7]. Infections in the temporal bone can lead to the inflammation of intracranial structures, such as the dura covering the lateral sinus. This can occur through a direct spread via the dehiscent bone, round or oval windows, endolymphatic sac, otic capsule fistula, or even through the cochlear aqueduct or perineural spaces in the internal acoustic meatus [8]. Bacterial spread through the intact bone can occur via retrograde thrombophlebitis in the small veins of the temporal bone, superior petrous sinus, cavernous sinus, carotid and tympanic tubes, periosteal venous plexus, and other potential vascular anastomoses [9]. Although not all infections result in venous thrombosis, this complication frequently occurs during otitis media in individuals with generalized hypercoagulability, which can be inherited or acquired [10].

Identifying symptoms of otogenic lateral sinus thrombosis is challenging. Ear-related issues, such as hearing impairment or ringing in the ears, are often more noticeable, whereas general symptoms, such as headaches or elevated body temperature, may indicate complications within the skull. In certain instances, prolonged symptoms associated with middle ear infections can result in complications. The emergence of neck swelling and/or stiff neck without evidence of otomastoiditis might suggest internal jugular vein thrombosis. While the “cord sign,” characterized by a hardened internal jugular vein along the sternocleidomastoid muscle’s front edge, is typically considered characteristic of this condition, it is not always evident. In this case, the voice changes preceded the diagnosis of vascular complications. The impairment of the vagus and glossopharyngeal nerves observed in our patient may have been due to inflammation around the nerves, caused by a thrombus extending to the jugular foramen and localized thrombophlebitis.

In such cases, contrast-enhanced computed tomography (CT) is the primary diagnostic modality. Although magnetic resonance venography (MRV) is not crucial for diagnosing vein thrombosis, it helps determine the extent of the thrombotic process and monitor the progress of anticoagulation therapy. Additionally, magnetic resonance imaging (MRI) scans were performed on patients showing clinical symptoms or CT findings suggestive of intracranial complications, as the MRI has proven to be more sensitive in detecting these issues.

This is the first reported case of JFS following a complicated acute otitis media in an adult patient. Involvement of the jugular foramen in this condition may be due to the direct spread of the clot from the adjacent lateral sinus. Another possible explanation is that inflammation around the vessel causes a vascular reaction in the jugular bulb, with the inflammation spreading along the vessel wall to the neck.

The recommended approach for managing thromboembolic events in adults typically involves administering low-molecular-weight heparin either intravenously or subcutaneously for several days, followed by cumulative oral anticoagulants until the prothrombin time reaches therapeutic levels, with a target of 2.5 [11]. Anticoagulant therapy should be discontinued once the risk factors for thrombotic events have been addressed, typically after a period of 3–6 months. In addition, appropriate antibiotic treatment must be administered. Steroid treatment should be considered when inner-ear involvement is present. In cases where surgery is required, infection is eliminated by draining the tympanomastoid cavity, which includes mastoidectomy and VT placement.

The limitations of this study include the restricted applicability of results derived from a single case report. Additionally, the credibility of the paper could be enhanced by addressing the possibility of overlooked diagnoses in comparable situations, owing to insufficient awareness.

## 5. Conclusions

JFS affecting the ear can elicit a range of otological manifestations that may be misinterpreted as middle ear pathology. A comprehensive assessment of the patient’s medical history and radiological imaging can facilitate the identification of the etiology of the symptoms, which may necessitate surgical or conservative management. Further investigation is imperative to obtain more extensive insights into the pathophysiology and therapeutic modalities of JFS affecting the ears.

## Data Availability

All data are available in the main text.
